# Changes in Sexual Function Following Uphold Transvaginal mesh Surgery for the Treatment of Urogenital Prolapse

**DOI:** 10.1038/s41598-019-52990-0

**Published:** 2019-11-19

**Authors:** Tsun-Wen Hsiao, Chin-Ru Ker, Kun-Ling Lin, Yung-Shun Juan, Ming-Ping Wu, Yi-yin Liu, Cheng-Yu Long

**Affiliations:** 1 Graduate Institute of Medicine, College of Medicine, Kaohsiung Medical University, Kaohsiung, Taiwan; 2 Department of Obstetrics and Gynecology, Kaohsiung Medical University Hospital, Kaohsiung Medical University, Kaohsiung, Taiwan; 3 Department of Urology, Kaohsiung Medical University Hospital, Kaohsiung Medical University, Kaohsiung, Taiwan; 4 Department of Obstetrics and Gynecology, Chi Mei Foundation Hospital, Tainan, Taiwan; 5 Department of Obstetrics and Gynecology, Kaohsiung Municipal Hsiao-Kang Hospital, Kaohsiung Medical University, Kaohsiung, Taiwan

**Keywords:** Outcomes research, Sexual dysfunction

## Abstract

Uphold transvaginal mesh implantation is an option for treating pelvic organ prolapse (POP). This prospective cohort study aims to evaluate the effect of Uphold transvaginal mesh implantation on female sexual function. 205 women with symptomatic POP were recruited and evaluated pre-operatively and re- evaluated six months post-operatively in terms of anatomical restoration, quality of life influenced by urinary incontinence and female sexual function. 30 women eventually completed the assessments and been statistically evaluated. The main outcome focused on sexual function. In our study, we found that Uphold transvaginal mesh surgery could achieve effective anatomical restoration of POP and better sexual function regardless of concomitant sling surgery.

## Introduction

Female pelvic organ prolapse (POP) can adversely affect the quality of life in regard to sexual function. Possible etiologic factors include nerve injury, blood supply impairment and changes in topography of pelvic floor. Pelvic surgery may also alter vaginal topography and affect subsequent sexual function. In addition, close proximity of vaginal grafts or scar tissue formation following pelvic surgery can cause stiffer vaginal wall inducing dyspareunia^1^. However, there are conflicting evidences with respect to the effect of transvaginal mesh (TVM) surgery on sexual function. Some studies showed TVM surgery resulted in deteriorating sexual function, while others revealed no or positive effect of the procedure on sexual function^2–11^. There are kinds of transvaginal mesh implantation methods such as single incision vaginal mesh and trans-obturator vaginal mesh. Different kinds of transvaginal mesh implantation may cause different effect on sexual function. Recent study has shown that single incision vaginal mesh may cause lower rate of mesh erosion than that of trans-obturator vaginal mesh^12^. Whether less erosion rate may relate to less sexual dysfunction or not need further investigation.

Uphold Lite Vaginal Support System with Capio SLIM Suture Capturing Device (Boston scientific, USA) is a newer generation of transvaginal mesh TVM kit. It has been used to treat POP since 2013. It is type I single incision mesh with characteristic of small in size and monoporous texture that could decrease biomaterial load to avoid mesh-related complications, such as mesh erosion. However, Food and Drug Administration (FDA) of the United States announced a warning in 2011 questioning the long-term safety of the surgical mesh. They have proposed on February 26, 2016 that urogynecologic surgical mesh instrumentation to be reclassified from class II to class III with special controls and be subjected to premarket notification requirements^13^. This warning raised the need to thoroughly evaluate the outcome of TVM. Few studies were conducted for Uphold TVM surgery including evaluation of post-treatment sexual function. Thus, we conducted this study to assess the effect of Uphold TVM surgery on sexual function.

## Result

Totally, 205 POP women were screened. Only 30 of them, who had completed the evaluation before and 6 months after surgery, were included in the study. Characteristics of all these subjects were listed in Table 1. The mean age was 57.3 ± 8.6 years old, mean parity was 2.5 ± 0.6 and mean BMI (kg/m^2^) was 25.2 ± 3.8. 6 of them were under hormone therapy, 26 women were menopausal, 13 of them had hypertension and 6 of them had a history of hysterectomy. All women had Uphold TVM surgery. Some of them had concomitant surgery included 5 posterior colporrhaphies, 9 vaginal hysterectomies (30%), 10 trans-obturator tapes (TVT-O) and 2 single-incision slings with Ophira (Promedon S.A, Argentina). The distribution of POP stage was listed in Table 2. All women suffered from symptomatic POP; 5 of them (16%) were POP stage 2; 22 of them (73%) POP stage 3; 3 of them (10%) POP stage 4; 25 of them (83%) advanced stage. Although only 30 out of 205 women had completed the whole evaluation, there were no significant difference of POP stage distribution between the 205 women group and 30 women group (Table 2).

**Table 1:** Clinical background of all subjects. Data are given as mean ± standard deviation, or n (%).

	N = 30
Mean age (years)	57.3 ± 8.6
Mean parity	2.5 ± 0.6
Mean BMI (kg/m^2^)	25.2 ± 3.8
Menopause	26 (86.7)
Hormone therapy	6 (20.0)
Diabetes Mellitus	0
Hypertension	13 (43.3)
History of hysterectomy	6 (20.0)
Uphold TVM	30
Concomitant surgery	
Posterior repair	5 (16.7)
Vaginal hysterectomy	9 (30.0)
TVT-O	10 (33.3)
Ophira	2 (6.7)

TVM, transvaginal mesh; BMI, body mass index; TVT-O, transobturator tape; TVT, tension-free vaginal tape.

**Table 2:** Pelvic organ prolapse quantification (POP-Q) stage distribution.

POP stage	N = 30	N = 205	*p* value
	number (%)	number (%)	
Stage 2	5 (16%)	18 (8.8%)	0.18^*^
Stage 3	22 (73%)	161 (80.5%)	0.52^*^
Stage 4	3 (10%)	26 (12.7%)	1.0^#^

^*^Chi-square test. ^#^Fisher’s exact test.

As for the POP-Q analysis, there was a significant improvement at points Aa, Ba, C, Ap, Bp and tvl (p < 0.001) after operation, and a 96.7% of success rate for POP correction was noted (Table 3). With respect to the changes in quality of life (QoL), there were statistically significant improvement in both UDI-6 and IIQ-7 questionnaires. No mesh erosion was found in all women at 6 months follow-up.

**Table 3:** Pelvic organ prolapse quantification (POP-Q) values and incontinence-related quality of life before and after surgery. Data are given as median (range) or mean ± standard deviation.

POP-Q parameters (cm)	N = 30		P value*
	Pre-op	Post-op	
Aa	1.0 (−1~3)	−1.5 (−3~−1)	<0.001*
Ba	3.0 (0~8)	−1.5 (−3~−1)	<0.001*
C	0 (−4~8)	8 (−9~−6)	<0.001*
Ap	−2 (−3~3)	−2 (−3~0)	<0.001*
Bp	0 (−2~7)	−2 (−3~0)	<0.001*
Tvl	9 (8~10)	10 (8~11)	<0.001*
UDI-6	22.2 ± 14.4	6.7 ± 3.3	<0.01**
IIQ-7	13.3 ± 6.2	6.2 ± 3.0	<0.01**

Pre-op, preoperative; Post-op, postoperative; Tvl, total vaginal length; UDI-6, Urogenital Distress Inventory; IIQ-7, Incontinence Impact Questionnaire. *Wilcoxon signed rank test; **Paired t-test.

We assessed female sexual function with FSFI questionnaire, and the total score and all domains except for lubrication domain showed significant improvement (Table 4). Subgroup analysis of FSFI comparing women with and without concomitant sling procedures revealed that both group showed significant improvement in all domains except for sexual desire. Subgroup statistical analysis between Uphold TVM only group and Uphold TVM plus midurethral sling group showed no difference in FSFI presentation in both groups (Table 5).

**Table 4:** Changes in scores of Female Sexual Function Index before and 6 months after surgery. Data are given as mean ± standard deviation.

Domains	N = 30		
	Pre-op	Post-op	P value*
Sexual desire	2.4 ± 0.9	2.9 ± 0.8	0.009**
Sexual arousal	2.8 ± 0.8	3.2 ± 0.5	0.011**
Lubrication	3.7 ± 1.2	3.8 ± 0.8	0.50
Orgasm	3.8 ± 1.1	4.1 ± 0.8	0.034**
Satisfaction	4.0 ±1.4	4.8 ± 0.9	<0.01**
Dyspareunia	4.3 ± 1.4	5.0 ± 1.0	<0.01**
Total scores	20.8 ± 5.3	23.7 ± 3.3	<0.01**

Pre-op, preoperatively; Post-op, postoperatively. *Paired t-test. **Statistical significance.

**Table 5:** Changes in scores of Female Sexual Function Index in both groups before and 6 months after surgery. Data are given as mean ± standard deviation.

Domains	Uphold alone (n = 18)			Uphold + sling (n = 12)		
	Pre-op	Post-op	P value*	Pre-op	Post-op	P value*
Sexual desire	2.5 ± 1.0^a^	3.3 ± 0.6	0.007**	2.3 ± 0.6^a^	2.3 ± 0.7	0.34
Sexual arousal	2.8 ± 1.0^b^	3.4 ± 0.5	0.044**	2.8 ± 0.5^b^	3.0 ± 0.4	0.025**
Lubrication	4.0 ± 0.9^c^	3.9 ± 0.6	0.59	3.1 ± 1.3^c^	3.6 ± 1.1	<0.001**
Orgasm	4.0 ± 1.3^d^	4.5 ± 0.6	0.07	3.4 ± 0.8^d^	3.5 ± 0.7	0.039**
Satisfaction	3.9 ± 1.6^e^	4.8 ± 1.0	0.001**	4.0 ± 0.9^e^	4.6 ± 0.7	<0.001**
Dyspareunia	4.3 ± 1.6^f^	5.1 ± 1.2	0.001**	4.3 ± 1.3^f^	4.8 ± 0.8	0.023**
Total scores	21.5 ± 6.3^g^	24.9 ± 3.3	0.009*	19.9 ± 3.3^g^	21.9 ± 2.4	<0.001**

Pre-op, preoperatively; Post-op, postoperatively.

p value comparing Uphold alone goupr and Uphold plus sling group: a= 0.44, b = 1.0, c = 0.054, d = 0.13, e = 0.85, f = 1.0, g = 0.37.

## Discussion

Current trend of evaluating the outcome of POP surgery has pivoted not only towards anatomic correction but also towards patients centered quality of life (QoL) and functional outcomes. Uphold Vaginal Support System with Capio SLIM Suture Capturing Device is a new single incision TVM kit widely used for managing POP. Its efficacy and effect on POP and QoL have been investigated in recent years^14^. Previous studies investigating influence of POP surgery on sexual function has revealed conflicting evidences^2–12^. These conflicting outcomes could be a result of difference in (1) various follow-up time periods between studies, (2) treatment response between premenopausal and postmenopausal women^10^, (3) mesh placement site in anterior, posterior or total vaginal wall^11^, or (4) routes and textures of mesh such as trans-obturator or single-incision mesh^12^. We applied this single incision transvaginal mesh in women, who had symptomatic POP.

In the aspect of anatomical restoration in women with POP-Q stage ≥2 apical prolapse, with or without concomitant anterior vaginal wall prolapse, Uphold TVM surgery could achieve optimal anatomical outcome at the vaginal apex. In our study, anatomical outcomes has significant improvement (96.7%) in all POP-Q points at 6-month follow-up (Aa, Ba, C, Ap, Bp, Tvl, p < 0.001). These objective outcomes also reflected in subjective outcome that UDI-6 and IIQ-7 questionnaire reported favorable results with significant improvement at 6-month follow-up (p < 0.01). These results were comparable with the previous study, in which Altman *et al*. reported a 94% success rate at 1-year follow-up and 83.3% at 5-year follow-up. With regards to subjective outcome, Altman, *et al*. also found 91% of the women at 1-year follow-up and 78.8% at 5-year follow-up experienced a significant decrease in bothersome sensation^3,4^. These data have shown the efficacy of Uphold transvaginal mesh surgery in anatomic restoration and associated symptoms.

Considering sexual function, our study has pointed out significant improvement in all domains of FSFI questionnaire including total score except for lubrication domain at 6-months follow-up. As compared to this result of the previous study, Altman, *et al*. reported significant decline in PISQ-12 scores (p < 0.001) in women who underwent apical prolapse correction with Uphold TVM at 1-year follow-up. More specifically in their study, the only significant changes were partner-related domains. In their study, dyspareunia improved and this was compatible with our result. However, the scores of other domains in PISQ-12 did not differ in the behavioral-emotive and physical sections. When it came to 5-year follow-up, the number of sexually active women became fewer but PISQ-12 including partner domain improved. These discrepancy between our study and previous studies may be explained by the difference in study subjects’ characteristics and their individual performance. FSFI questionnaire doesn’t include partner domain but only demonstrates female’s aspect of sexual function. The improvement in sexual function may mainly benefit female but not male partners in short-term period. Dyspareunia improved in both studies which can explained this interpretation as well.

Dyspareunia is a concern related to sexual function and transvaginal mesh. Different types of transvaginal mesh may have different results in sexual function. Uphold Vaginal Support System is designed for single incision surgery that may express less mesh extrusion thus less dyspareunia. Lo *et al*. reported that single incision mesh achieved no mesh exposure event and significant improvement in PISQ-12 in short term follow-up^5^. Long *et al*. compared the extrusion rate between single incision mesh and trans-obturator mesh approaches and revealed less extrusion and dyspareunia rate in single incision mesh. In their study, they reported lower score in dyspareunia domain in FSFI questionnaire following trans-obturator TVM repair, and mesh extrusion rates as 11.4% at 4 and 10 weeks postoperatively. Dyspareunia has been one of the concerns associated with the use of trans-obturator synthetic mesh, ranging from 20–36% in previous studies^12^. Different from trans-obturator mesh, we found significant improvements on all domains of FSFI after single-incision TVM surgery, except for the lubrication domain. No mesh extrusion was found in our study. This improvement in dyspareunia and sexual function may be due to the evolution of new TVM, that is smaller in size, lighter, less dense texture, and single-incision design. Another reason for favorable sexual function may be that only anterior compartment but not total vaginal wall was involved in this Uphold TVM procedure. In our previous study, total TVM appeared to cause a greater sexual impairment compared with anterior TVM alone^11^.

There are some limitations in our study. We evaluated the subjects before and 6 months after treatment according to previous literatures. However, in this interval of follow-up, we could only reveal short-term effect of the intervention. Thirty of the 250 women had completed the evaluation before and 6 months after surgery. The remaining 175 women could not complete the study, mainly due to resistance of completing questionnaires because of time-consuming and being ashamed of answering personal questions. Due to relatively small number of cases, we use multiple parameter POP-Q system to evaluate the postoperative changes so to have better statistical power. Although there were some characteristics of our patients, we tried not to make any change to the patient’s underlying conditions such as medication of controlling hypertension, topical use of hormone therapy for a long time. Application of topical estrogen cream in treating postmenopausal women may have an effect on sexual function^10,15,16^. In our series, 6 women had hormone therapy. Very low-dose topical vaginal estrogen hormone was used in 5 cases (5/6; 83.3%) and systemic low-dose hormone was used by one patient (1/6; 17.7%). They kept hormone therapy long before, during and after POP surgery, we didn’t change the patient’s usual management to avoid adding another possible variable. We treat patients with only one kind of transvaginal mesh to keep consistent management. Some concomitant surgeries were inevitable because it is clinically common to find multiple problems to be solved in one patient. It is estimated 50% patient with POP may have stress incontinence at the same time. All concomitant surgeries were performed on the basis of patient’s consents after fully discussion. Several studies have shown that vaginal hysterectomy would not be a factor interfering sexual function^3,17,18^. We also did the subgroup comparison between women treated with Uphold TVM with and without concomitant mid-urethral sling, the statistical analysis shows no difference between two groups which indicated that mid-urethral sling may not be the factor to change sexual function in our study. Considering limitation mentioned, more rigorous study is needed in the future.

In this study, focusing on the effect of Uphold TVM surgery on sexual function. The results of our study indicated that the Uphold TVM surgery was effective in anatomical restoration of POP and was favorable for sexual function. Women with Uphold TVM surgery reported higher scores of FSFI in most domains, except for the lubrication domain. Moreover, no mesh extrusion is found and there is no detrimental effect on sexual function in women treated with Uphold TVM with or without sling surgery. We believe these concepts are helpful when counseling women with POP before surgery arrangement about possible impacts on sexual function. However, we acknowledge that the case numbers and duration of follow-up of this study were relatively limited and short, studies with larger sample size and longer investigation are expected in the future.

## Material and Methods

### Inclusion and exclusion criteria

From September 2015 through August 2017, two hundred and five women with symptomatic POP stages ranging from II to IV defined by the POP quantification (POP-Q) staging system^11^ were treated with TVM with Uphold TVM at our hospitals performed by two skilled and experienced urogynecologic physicians. These women were fully informed and discussed about choices of surgeries. Only women signed the inform consents of surgery and participation in this study before treatment started were included in the study.

“Sexually active” was defined as having vaginal intercourse at least 1 time per month within 6 months before treatment and continuously after treatment. Inclusion criteria were women who agree to participate in the study, who were POP stage from II to IV initially and undergoing transvaginal mesh surgery with Uphold TVM, who were sexually active and who completed evaluation pre-operatively and 6 months post-operatively. Exclusion criteria were women did not agree to participate in this study, women who cannot complete the whole treatment and evaluation. These patients were interviewed using short standardized forms including Urogenital Distress Inventory (UDI-6), Incontinence Impact Questionnaire (IIQ-7) and Female sexual function index Questionnaire (FSFI) concerning their urinary and sexual symptoms before and 6 months after treatment. Primary outcome was the POP-Q, UDI-6, IIQ-7 and FSFI. Anatomical restoration was evaluated before and 6 months after treatment by pelvic examination based on POP-Q system postoperatively and surgical failure was defined as the most distal portion being POP stage II or greater, regardless of a primary or a new site^19,20^.

Concomitant operations included posterior colporrhaphy, vaginal total hysterectomy, midurethral sling with TVT-O (Gynecare TVT-Obturator System, Ethicon, Inc., Somerville, NJ, USA) and mini-sling (Ophira Mini Sling System; Promedon, Córdoba, Argentina). Midurethral sling were performed in some women with stress incontinence or occult urodynamic stress incontinence (USI), unless they did not agree to have additional surgery.

### Surgical procedure

We applied Uphold Lite Vaginal Support System with Capio SLIM Suture Capturing Device (Boston scientific, USA) in the anterior vaginal wall. Diluted pitressin (Pfizer, New York, USA) with 100 ml 9% normal saline was injected to anterior vaginal wall below the bladder neck. Initial incision site was made 3 cm below urethral orifice till 2 cm above uterine cervix. Compartment between anterior vaginal wall and bladder visceral layer was dissected by metzenbaum scissors bilaterally. Check bilateral ischial spine manually with index finger. Capio SLIM Suture Capturing Device was assembled with mesh. Insert the tip of assembled device with mesh guided by index finger to ischial spine. The surgeon passed the special designed needle through the sacrospinous ligament at a level of 2 cm medial to the ischial spine, anchoring the mesh on the sacrospinous ligament. Repeated the procedure with the same manner on the other site. Then we sutured the mesh with the cervical stroma, bilateral cardinal ligaments, and pubocervical fascia beneath bladder neck with PDS 2.0 (Ethicon, New Jersey, USA). Finally, closure of the vaginal incision wound was made with vicryl 2.0 (Ethicon, New Jersey, USA) continuously. Vaginal packing was placed for 48 hours after surgery.

Ethics approval by the Institutional Review Board of Kaohsiung medical university hospitals had been obtained for collecting data and further analysis. All participants were requested to sign an informed consent approved by the Institutional Review Board of the Kaohsiung Medical University before the treatment. Surgery was performed on the basis of official surgery consent in Kaohsiung Medical University hospital. Statistical analysis was performed using Paired t-test or Wilcoxon signed-rank test for continuous variables. A difference was considered statistically significant when  p< 0.05. The study protocols were approved by the Institutional Review Board of Kaohsiung Medical University Hospital, by which relevant guidelines and regulations were followed accordingly.

We assessed the power of tests for differentiating the surgical outcome in this study. Power analysis showed that around 40 to 50 cases would have a power of 80%. Some comparisons could not reach sufficient statistical power with limited number of cases in our series. We used multiple parameters of POP-Q system to evaluate the postoperative changes and found that more than 30 women included in this study, there would be a power of more than 85% for discrimination.

### Ethical approval and informed consent

Ethics approval by the Institutional Review Board of Kaohsiung Medical University Hospital had been obtained for data analysis.

## Data Availability

The datasets analyzed for the current study are available from the corresponding author upon reasonable request.
